# Ten years with biosimilar rhGH in clinical practice in Sweden – experience from the prospective PATRO children and adult studies

**DOI:** 10.1186/s12902-020-0535-4

**Published:** 2020-04-29

**Authors:** Elena Lundberg, Berit Kriström, Hichem Zouater, Anna Deleskog, Charlotte Höybye

**Affiliations:** 1 Institute of Clinical Science/Pediatrics, Umeå University, SE-90185 Umeå, Sweden; 2 Sandoz Biopharmaceutical, c/o HEXAL AG, Industriestr. 25, D-83607 Holzkirchen, Germany; 3 Sandoz A/S, Copenhagen, Denmark; 4 Department of Molecular Medicine and Surgery, Karolinska Institute, Stockholm, Sweden

**Keywords:** Recombinant growth hormone, Safety, Effectiveness, Antibodies, Omnitrope

## Abstract

**Background:**

In 2007, Omnitrope® was the first biosimilar recombinant human growth hormone (rhGH) to be approved in Sweden for treatment in adults and children. Over 10 years’ safety and effectiveness data for biosimilar rhGH can now be presented.

**Methods:**

PATRO Children and PATRO Adults are multicenter, longitudinal, observational, post-marketing surveillance studies. Eligible patients include children 0–18 years and adults receiving biosimilar rhGH treatment. Adverse events (AEs) are monitored for safety evaluation. Growth variables in children and metabolic data in adults are recorded for effectiveness evaluation.

**Results:**

As of January 2019, data from 136 children (48% male) were reported from Swedish centers. Mean age in rhGH treatment-naïve patients at study entry (*n* = 114) was 7.5 years, with mean 3.6 years treatment duration. No severe AEs of diabetes, impaired glucose tolerance, or malignancy were reported. The most frequently reported AE was nasopharyngitis (*n* = 16 patients). No clinically relevant anti-hGH or neutralizing antibodies were observed. The mean change from baseline in height standard deviation score (SDS) in naïve prepubertal GH deficiency patients was + 0.79 at 1 year, + 1.27 at 2 years, and + 1.55 at 3 years. Data from 293 adults (44% rhGH-naïve, 51% male) were included. Fatigue was the most frequently reported AE (*n* = 26 patients). The incidence of new neoplasms or existing neoplasm progression was 23.8 patients per 1000 patient-years. Type 2 diabetes mellitus was reported in four patients. At baseline in rhGH-naïve adults, mean (SD) body mass index (BMI) was 29.1 (5.6) kg/m^2^ and mean (SD) insulin-like growth factor (IGF)-I SDS was − 3.0 (1.4). Mean daily dose increased from 0.1 mg at baseline to 0.3 mg after 4 years. IGF-I SDS normalized during the first year of treatment. Mean BMI and glucose were unchanged over 4 years, while low−/high-density lipoprotein cholesterol ratio decreased.

**Conclusions:**

For the first time, Swedish data from the PATRO Children and Adults studies are presented. The 10-year data suggest that biosimilar rhGH is well tolerated across pediatric and adult indications. Safety and effectiveness were similar to previous reports for other rhGH preparations. These results need to be confirmed in larger cohorts, highlighting the importance of long-term post-marketing studies.

## Background

Growth hormone (GH) replacement therapy has been used in clinical practice for more than 50 years. Initially, only children with GH deficiency (GHD) were treated due to limited availability of the hormone. The development of recombinant human GH (rhGH) increased supply, leading to approval of rhGH therapy in other pediatric indications associated with poor growth [1]. In adults, GH replacement therapy has been used since the 1990s, with the goal of improving metabolic and psychosocial impairments associated with GHD [2].

Biosimilar rhGH (Omnitrope®, Sandoz, Kundl, Austria) was approved in Australia in 2004, and by the European Medical Agency (EMA) in 2006, as the world’s first biosimilar rhGH, and has been used in Sweden for over 10 years.

Like all active biological medicines, biosimilar rhGH could induce an immune response with a potential risk that anti-rhGH or neutralizing antibodies may occur during treatment. This has raised some safety and efficacy concerns [3–5]; to ensure safe and effective use of biosimilar medicines, the EMA demands high-quality scientific evidence [6, 7]. To meet the EMA biosimilar pharmacovigilance requirements, the multicenter, longitudinal, non-interventional, observational PATRO (Patients TReated with Omnitrope®) studies were designed to evaluate the long-term safety and effectiveness of biosimilar rhGH in clinical practice [8, 9]. Enrollment of Swedish patients in PATRO Children started in 2007 with only few patients registered before 2009; enrollment in PATRO Adults started in 2011.

Here, we present Swedish data from the PATRO Children and Adult databases, focusing on safety, particularly the development of diabetes mellitus and malignancies during the study treatment period. In addition, changes in height variables in children and metabolic effects in adults are reported. Swedish data collected before 2016 were included in previous international reports but the present report includes data for an additional 3 years and more detailed analyses. 

## Methods

### Study design and patient population

The primary aim of the PATRO Children (patients aged 0–18 years) and Adults studies is to collect and analyze data on the long-term safety of biosimilar rhGH in patients treated in routine clinical practice. The primary objective is to follow patients for safety concerns, such as the occurrence of diabetes mellitus or malignancies, and to detect anti-hGH antibodies inducing a lack or loss of effectiveness in pediatric patients. The secondary aims are to assess long-term treatment effectiveness in terms of growth response in children, and the effects on body composition and cardiovascular risk factors in adults.

The designs of both studies have previously been described in detail [8, 9]. Briefly, children and adults who are receiving treatment with biosimilar rhGH, both rhGH-naïve and those previously treated with another rhGH medicine, are eligible for inclusion. Patients were enrolled consecutively and no formal exclusion criteria were applied. The frequency of visits follows routine clinical practice; no additional visits, tests, or assessments are required as part of the study. In Sweden all patients fulfilling the diagnostic criteria for GHD and without contraindications are offered GH treatment. Biosimilar rhGH dosing is at the discretion of the treating physicians as reported to the PATRO database; information on dose is collected at baseline (entry into the PATRO study) and at least yearly thereafter.

### Safety assessments

All adverse events (AEs) and serious AEs (SAEs) were collected, recorded in electronic case report forms (eCRF), and entered into the sponsor’s safety database for the duration of biosimilar rhGH treatment.

### Growth evaluation in children

Height and weight were measured at each visit and converted to standard deviation scores (SDS) using the current Swedish growth reference, according to sex and age [10]. The outcome variables used for evaluation of effectiveness were prepubertal first year gain in height SDS (ΔHSDS) (and if possible due to age of the child, also ΔHSDS 0–2 and 0–3 years) in rhGH-naïve patients. The calculation of prepubertal HSDS included only data from girls before reaching 10 years of age and from boys before reaching 11 years [11], in order to exclude interference of the pubertal component in the combined average growth reference.

Investigator assessment was used to confirm if patients had reached adult height (AH) or near AH. Attained AH in centimeters was converted to adult HSDS at age 18 years, even if AH was reached at an earlier age. Body mass index (BMI) SDS was derived from our Swedish reference [12]. Puberty stage was assessed clinically by the investigator, according to standards established by Tanner [13] and Prader [14].

### Metabolic evaluation in adults

BMI, waist circumference, blood lipids, and fasting blood glucose were assessed at treatment start and yearly thereafter during 4 years of treatment.

### Laboratory analyses

Anti-hGH antibody determination in children was carried out upon request in routine clinical practice or as requested by the EMA in rhGH-naïve children until 2 years after the start of biosimilar rhGH treatment. Analyses were performed at a single selected laboratory (Q^2^ Solutions, Valencia, CA, USA). Results were entered according to the sample collection date. Antibodies were measured by using a semi-quantitative radioimmunoassay. Briefly, radio isotope labeled rhGH (125I-rhGH) was incubated at a refrigerated temperature overnight with the serum specimen; antibodies in the specimen bind to the 125I rhGH. The bound/free separation was achieved with 20% polyethylene glycol 8000 and centrifugation. The radioactivity of the pellet remaining in the assay tube was counted in a gamma counter and the response was proportional to the amount of anti-rhGH antibodies present in the specimen. The results were reported in index units calculated from a normal pool. Specimens were considered positive when the index value was > 1.76.

Insulin-like growth factor (IGF)-I μg/L was measured locally at each participating center and SDS values were calculated according to laboratory-performed analyses. Serum high-density lipoprotein (HDL) cholesterol was measured at a local laboratory, and serum concentrations of low-density lipoprotein (LDL) cholesterol were calculated using Friedewald’s formula [15].

### Data collection and statistical analyses

Available auxological data, biochemistry, medical history, and concomitant therapy were recorded in the eCRF. The safety analysis set (SAF) included all patients who had any data documented in the eCRF and who received at least one dose of biosimilar rhGH. The effectiveness analysis set (EFF) is a subset of the SAF and included all patients with a documented height measurement at the start of biosimilar rhGH treatment (baseline) and at least one measurement of height during treatment, at least 60 days after baseline.

Statistical methods included routine descriptive statistics. Continuous parameters (e.g. height, weight, BMI) were presented as the n, mean, and standard deviation (SD). Categorical parameters (e.g. adverse events) were presented as frequency and percentage. The incidence of an AE within a time period was defined as the number of patients who experienced the event divided by the number of patient-years. Patient-years were calculated by period, with each patient contributing only the actual time they were under observation in this period. A bar chart was generated for the proportion and number of naïve and pre-treated Swedish patients. Box plots were generated for rhGH dose and height-derived parameters (e.g. change from baseline in HSDS). Box plots were displayed by re-aligned visits, and pre-treatment and indication status. A time-series graph was generated for height-derived parameters (e.g. HSDS) over 3 years by indication. For effectiveness results, SDS were generated using the relative deviation from the mean value of normally growing children of the same gender and chronological age. Statistical analyses for this study were performed using the software SAS 9.4. The current interim analysis was performed in January 2019 for both studies.

## Results

### PATRO children

#### Baseline characteristics

**Fig. 1:**
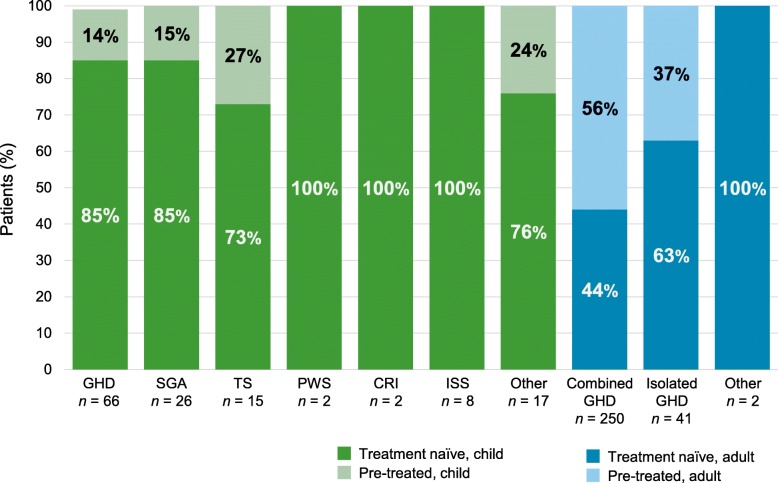
Proportion and number of naïve and pre-treated Swedish patients from the safety population in the PATRO Children and PATRO Adults studies**.** *CRI* chronic renal insufficiency, *GHD* growth hormone deficiency, *ISS* idiopathic short stature, *PWS* Prader–Willi syndrome, *SGA* small for gestational age, *TS* Turner syndrome

As of January 2019, 136 patients (65 males [48%] and 71 females [52%]), had been enrolled in PATRO Children from 9 sites in Sweden. At the time of analysis, 87 patients (64%) were still active in the study. The indications for rhGH treatment were: GHD, *n* = 66 (49%); SGA, *n* = 26 (19%); TS, *n* = 15 (11%); PWS, *n* = 2 (2%); CRI, *n* = 2 (2%); idiopathic short stature (ISS), *n* = 8 (6%); and short stature due to other reasons, *n* = 17 (13%) (Fig. 1). Data from the GHD, SGA and TS groups were used for the EFF; effectiveness data from the groups with PWS, CRI and ISS are not presented due to low patient numbers in the study. However, data from all patients are included in the SAF. Overall, 114 patients (84%) were naïve to rhGH therapy at study entry, 21 patients (15%) had previously received rhGH treatment, and for 1 patient pre-treatment information was missing.

Overall, 45 patients (33%) completed 5 years of treatment. Mean (range) age at treatment start for rhGH-naïve patients was 7.5 years (0.6–17.2); 7.7 (0.6–16.9) years for males and 7.2 (1.5–17.2) years for females. Mean (SD) duration of treatment was 41.9 (33.5) months for rhGH-naïve patients (~ 3.5 years). In pre-treated patients, the mean (range) age at start of biosmilar rhGH therapy was 10.6 years (5.9–15.6); 10.2 (7.4–15.0) years for males and 10.8 (5.9–15.6) years for females. Mean (SD) duration of rhGH treatment was 56.9 (34.1) months for pre-treated patients (~ 4.7 years).

#### Safety

##### Adverse events

Since the study began in 2007, no drug-related SAEs of diabetes mellitus, impaired glucose tolerance, or new malignancy have been reported. In total, 47 patients (35%) have reported 121 AEs, with only two AEs in one patient (1%) suspected to be related to treatment. Overall, 20 events in 11 patients (8%) were considered serious, but not treatment related. The most frequent AEs are presented in Table 1***.*** The AE with the highest incidence was nasopharyngitis (*n* = 16 patients [12%]; incidence 31.7 patients per 1000 patient-years), followed by ear infection, varicella, and viral infection (*n* = 5 patients each [4%]; incidence 9.9 patients per 1000 patient-years; Table 1).

**Table 1:** Most commonly reported adverse events in PATRO Children (incidence > 7.0) and PATRO Adults (incidence > 10.0)

MedDRA dictionary preferred term	Maximum intensity				Total patients ***n*** (%)	Incidence^**a**^
	Mild ***n*** (%)	Moderate ***n*** (%)	Severe ***n*** (%)	Missing ***n*** (%)		
**PATRO Children**						
Nasopharyngitis	13 (9.6)	3 (2.2)	0 (0.0)	0 (0.0)	16 (11.8)	31.70
Ear infection	1 (0.7)	4 (2.9)	0 (0.0)	0 (0.0)	5 (3.7)	9.91
Varicella	2 (1.5)	2 (1.5)	0 (0.0)	1 (0.7)	5 (3.7)	9.91
Viral infection	4 (2.9)	1 (0.7)	0 (0.0)	0 (0.0)	5 (3.7)	9.91
Headache	1 (0.7)	3 (2.2)	0 (0.0)	0 (0.0)	4 (2.9)	7.93
Pneumonia	1 (0.7)	3 (2.2)	0 (0.0)	0 (0.0)	4 (2.9)	7.93
Vomiting	3 (2.2)	1 (0.7)	0 (0.0)	0 (0.0)	4 (2.9)	7.93
**PATRO Adults**						
Fatigue	20 (6.8)	6 (2.0)	0 (0.0)	0 (0.0)	26 (8.9)	23.82
Arthralgia	13 (4.4)	3 (1.0)	0 (0.0)	1 (0.3)	17 (5.8)	15.58
Headache	9 (3.1)	4 (1.4)	0 (0.0)	0 (0.0)	13 (4.4)	11.91
Nasopharyngitis	11 (3.8)	2 (0.7)	0 (0.0)	0 (0.0)	13 (4.4)	11.91
Dizziness	9 (3.1)	1 (0.3)	2 (0.7)	0 (0.0)	12 (4.1)	11.0
Neoplasm progression	1 (0.3)	4 (1.4)	7 (2.4)	0 (0.0)	12 (4.1)	11.0
Osteopenia	12 (4.1)	0 (0.0)	0 (0.0)	0 (0.0)	12 (4.1)	11.0
Edema peripheral	7 (2.4)	3 (1.0)	1 (0.3)	0 (0.0)	11 (3.8)	10.08

^a^Incidence is defined as the number of patients with respective AE per 1000 patient-years; PATRO Children patient-years = 504.7, PATRO Adults patient-years = 1091.3

*AE* Adverse event, *PATRO* Patients TReated with Omnitrope®

##### Immunogenicity

In total, 86 samples from 40 rhGH-naïve patients were assessed for anti-hGH antibodies. Only one positive anti-hGH antibody titer occurred transiently in a treatment-naïve patient prior to rhGH treatment; all subsequent results for this patient were negative.

##### Treatment discontinuation

At the time of the interim analysis, 49 of the 136 patients (36.0%) had discontinued treatment; 26 males and 23 females. Reasons for treatment discontinuation included: reaching AH/bone age maturation (*n* = 18); reaching near AH (*n* = 5); patient satisfaction with current height (*n* = 1); referral to an adult clinic for continued rhGH therapy (*n* = 5); and lost to follow up (*n* = 3);

Three patients discontinued enrollment due to the decision of their treating physician. One male patient had a complicated psychosocial situation after surgery for craniopharyngiomas, resulting in various thyroxine, cortisone and antidiuretic hormone therapy, although he continued with biosimilar rhGH treatment. The remaining two patients were not started on rhGH treatment for other reasons.

Five patients were non-compliant (three chose to stop rhGH treatment after a period of low treatment compliance, and a further two changed to another rhGH brand due to personal preference for the “old” injection pen).

Six patients were non-responders and discontinued treatment as a result. The first was a male diagnosed with familial short stature and suspected fetal alcohol syndrome who was treated for 18 months from age 11 years. First year ΔHSDS was + 0.3 with an rhGH dose 0.050 mg/kg/d. The second non-responder was a female diagnosed with hypochondroplasia, who was treated for 41 months from age 6.4 years with an rhGH dose of 0.036 mg/kg/d. First year ΔHSDS was + 0.16. IGF-I at baseline was missing, but was reported as 346 μg/L at the Year 1 visit. The remaining four non-responders (one TS, two SGA, and one GHD with an unspecified congenital genetic syndrome) had low growth response but had been treated with rhGH doses below those recommended in the label for reasons that were not reported (but not AE-related).

#### Changing to biosimilar rhGH from other rhGH brands

Changing treatment to biosimilar rhGH in children diagnosed with GHD (*n =* 9), TS (*n =* 4), and SGA (*n =* 4) was not associated with any safety issues. The proportion of patients with non-serious, non-drug-related AEs was similar in naïve (32% [*n =* 36/114]) and pre-treated (38% [*n =* 8/21]) patients.

#### Effectiveness

##### rhGH dose

**Fig. 2:**
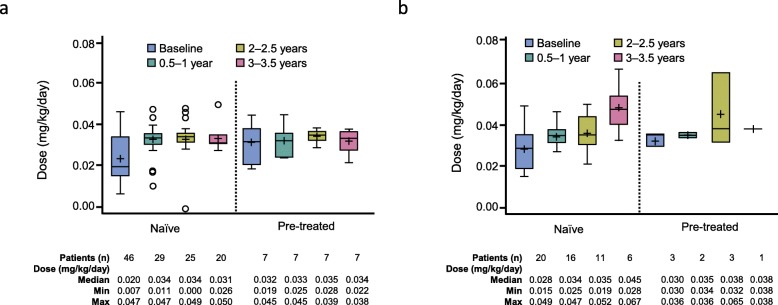
rhGH dose per 6-month period by indication and pre-treatment status for GHD (**a**) and SGA (**b**) patients in the safety population enrolled in PATRO Children. *GHD* growth hormone deficiency, *rhGH* recombinant human growth hormone, *SGA* small for gestational age

Mean rhGH dose over 2 years is presented in Fig. 2 for GHD and SGA patients. In all diagnosis groups, rhGH doses were lower at baseline than at 0.5 years. For rhGH-naïve GHD patients at baseline, median dose was 0.020 mg/kg/d and increased to 0.034 mg/kg/d at 0.5–1 year. For the rhGH-naïve SGA group, median starting dose was 0.028 mg/kg/d and increased to 0.034 mg/kg/d at 0.5–1 year. For the rhGH-naïve TS group, mean baseline dose was 0.030 mg/kg/d and increased to 0.042 mg/kg/d at 0.5–1 year.

##### Height assessment

**Fig. 3:**
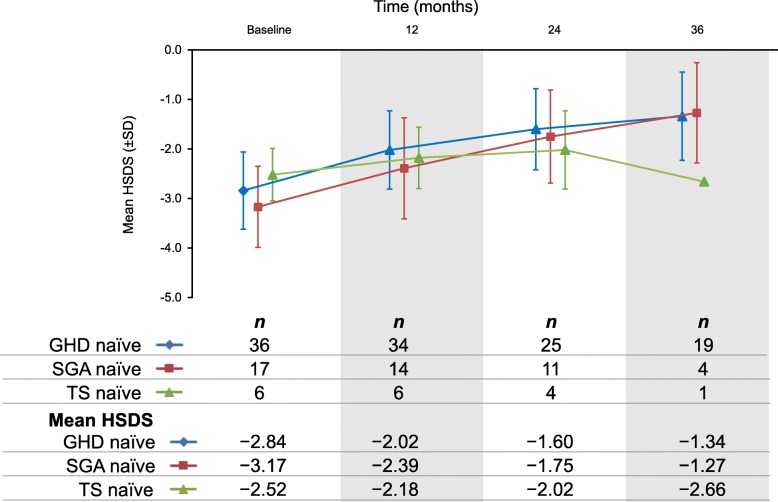
HSDS of biosimilar rhGH treatment over 3 years for pre-pubertal rhGH treatment-naïve GHD, SGA, and TS patients who are younger than 10 years (girls) or 11 years (boys). *GHD* growth hormone deficiency, *HSDS* height standard deviation score, *rhGH* recombinant human growth hormone, *SD* standard deviation, *SGA* small for gestational age, *TS* Turner syndrome

**Fig. 4:**
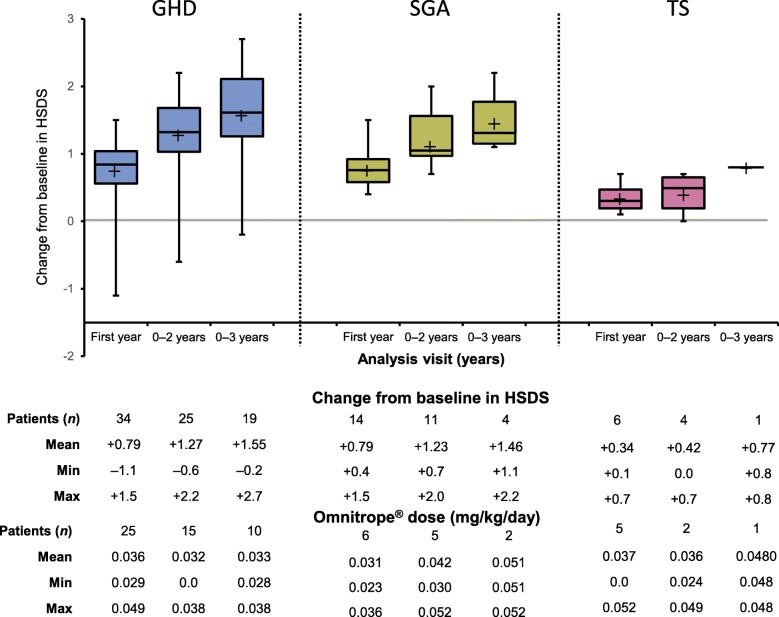
Change in HSDS from baseline by indication for treatment-naïve, pre-pubertal patients that are younger than 10 years (girls) or 11 years (boys). *GHD* growth hormone deficiency, *HSDS* height standard deviation score, *SGA* small for gestational age, *TS* Turner syndrome

Attained HSDS from baseline to 3 years of treatment in rhGH-naïve prepubertal patients diagnosed with GHD, SGA and TS is presented in Fig. 3. The change in HSDS from baseline for rhGH-naïve prepubertal patients with GHD, SGA and TS is shown in Fig. 4. In GHD patients, mean change in HSDS from baseline (ΔHSDS) was + 0.79 at 0–1 year, + 1.27 at 0–2 years, and + 1.55 at 0–3 years. For SGA patients, ΔHSDS was + 0.79 at 0–1 year, + 1.23 at 0–2 years, and + 1.46 at 0–3 years. For TS patients, ΔHSDS was + 0.34 at 0–1 year, + 0.42 at 0–2 years, and + 0.77 at 0–3 years (when only 1 TS patient remained in the study)*.*

Overall, at the time of analysis, only 11 rhGH-naïve patients had reached AH. Four male GHD patients had mean AHSDS of − 1.9 and one female GHD patient had AHSDS of − 2.0. Two female TS patients had mean AHSDS of − 2.1. One male patient born SGA had AHSDS of − 2.9 and a female patient had AHSDS of − 2.6. One male patient with ISS had AHSDS of − 2.5. In the remaining patient, AHSDS was not recorded.

### PATRO adults

#### Baseline characteristics

As of January 2019, 293 patients had been enrolled in PATRO Adults from 6 sites in Sweden. There were 148 male patients (51%) and 145 female patients (50%); 130 patients (44%) were rhGH naïve at study entry and 163 (56%) were pre-treated. Mean (SD) age was 50.0 (15.4) years for rhGH-naïve patients and 54.0 (15.1) years for pre-treated patients. Of 250 patients with combined GHD (multiple pituitary deficiencies), 102 patients (41%) were rhGH naïve and 148 patients (59%) were pre-treated (Fig. 1). Isolated GHD was observed in 41 patients, of whom 26 (63%) were rhGH naïve and 15 (37%) were pre-treated.

The total cohort comprised 250 patients with adulthood-onset GHD and 43 patients with childhood-onset GHD. In the adulthood-onset group, 35 patients had isolated GHD and 211 had combined GHD. The number of additional pituitary deficiencies were as follows: one, 46 (22%) patients; two, 57 (27%) patients; three, 92 (44%) patients; and four, 16 (8%) patients. In the remaining two the diagnosis was entered as ‘other’. In the childhood-onset group, 37 had combined GHD and 6 had isolated GHD. The most common additional pituitary deficiencies were thyroid-stimulating hormone deficiency (*n =* 210; 84%), follicle-stimulating hormone/luteinizing hormone deficiency (*n =* 195; 78%), and adrenocorticotropic hormone deficiency (*n =* 147; 59%).

#### Safety

##### Adverse events

In total, 218 patients (74%) reported 752 AEs. AEs were mild in 169 patients, moderate in 115 patients, and severe in 33 patients. SAEs were reported in 102 patients (35%, *n =* 185 events); these were mild in 34 patients, moderate in 60 patients, and severe in 33 patients.

The most frequently reported AEs are presented in Table 1. Fatigue was the most commonly reported AE, occurring in 26 patients (9%; incidence 24 patients per 1000 patient-years).

Development of new neoplasms or reoccurrence/progression of a pre-existing neoplasm (*n =* 32 events) was reported in 26 patients (11 females and 15 males), of whom three developed two different neoplasm types. Four of the patients had isolated GHD and 22 had combined GHD. The neoplasms developed after a median (range) treatment duration in the study of 26.1 (1.3–82.5) months. Of the 26 patients (incidence 23.8 patients per 1000 patient-years), 8 patients had malignant neoplasms, 16 patients had benign neoplasms, 1 patient had one malignant and one benign neoplasm, and 1 patient was missing malignancy information. The benign neoplasms were in most cases progression of a residual pituitary tumor. Of the malignant neoplasms, one female patient had breast cancer, one female patient had a malignant brain tumor, one female patient had progression of a brain tumor; one male patient had lung cancer, one male patient had renal cancer, one male patient had metastatic lymph nodes, and four male patients had prostate cancer. In 13 of the patients (*n =* 15 events), the rhGH dose was not changed, in seven patients (*n =* 7 events) rhGH treatment was permanently discontinued, in 7 patients (*n =* 9 events) the rhGH dose was interrupted, and in one patient (*n =* 1 event) the rhGH dose was reduced. The development of these neoplasms was considered unrelated to rhGH treatment in all cases.

Onset of diabetes mellitus type 2 after rhGH start was reported in four patients (2 females and 2 males, 0.4%; incidence 3.7 patients per 1000 patient-years). One of the patients had isolated GHD and the other three had combined GHD. Diabetes mellitus was diagnosed after 65.9, 8.6, and 32.8 months, respectively, in three patients. In two of these patients, the rhGH dose was not changed, and in one patient rhGH treatment was permanently discontinued. None of the cases of diabetes mellitus onset were considered related to rhGH treatment. In one patient who had previously received rhGH treatment for 18.6 years, worsening of previously known diabetes occurred on the first day of biosimilar rhGH treatment. In accordance with the patient’s request, rhGH treatment was consequently interrupted. Given that the deterioration occurred shortly after biosimilar rhGH treatment started, this event was considered treatment related.

Drug-related AEs were reported in 26 patients (9%; *n =* 35 events) and drug-related SAEs in 6 patients (2%; *n =* 6 events). Drug-related AEs comprised: peripheral edema (*n =* 7 patients); headache (*n =* 4 patients); myalgia (*n =* 4 patients); arthralgia (*n =* 3 patients); arthritis (*n =* 2 patients); and bone lesion, carpal tunnel syndrome, diabetes mellitus, diarrhea, impaired fasting glucose, increased blood glucose, joint stiffness, nausea, pain in extremity, induration, edema, peripheral swelling, and prescribed overdose (*n =* 1 each). Most of these AEs were mild, transient, and related to the rhGH dose. Drug-related SAEs included arthralgia, arthritis, bone lesion, carpal tunnel syndrome, diabetes mellitus and peripheral edema (*n =* 1 each).

##### Treatment discontinuation

In total, 47 patients (16%) discontinued treatment (20 males, 27 females). Of these, 17 patients (6 males, 11 females) discontinued treatment due to an AE, 6 patients (4 males, 2 females) switched to another rhGH treatment, 6 patients (3 males, 3 females) discontinued because they were referred to another clinic, 10 patients (4 males, 6 females) did not want to continue rhGH treatment, 6 patients (2 males, 4 females) discontinued for other reasons, one female patient discontinued due to non-compliance, and one male patient was lost to follow up.

#### Effectiveness

Effectiveness parameters for rhGH-naïve and pre-treated adults are shown in Table 2. Mean (SD) IGF-I SDS was − 3.0 (1.4) at baseline in rhGH-naïve patients and normalized during the first year of rhGH treatment. At baseline, patients had a high waist circumference; (mean [SD]: 100.6 [13.6] cm) and were overweight (mean [SD] BMI 29.1 [5.6] kg/m^2^); no changes in these variables were observed during rhGH treatment. Mean (SD) rhGH dose increased from 0.1 (0.1) mg/day at the start of treatment to 0.3 (0.2) mg/day at the 4-year visit. Mean LDL/HDL cholesterol ratio was 3.1 (1.4) mmol/L at baseline, and decreased to 2.2 (0.6) mmol/L at the 4-year visit Table 2. No change in fasting blood glucose was seen 6.1 (2.7) mmol/L at baseline and 6.0 (1.9) mmol/L following 4 years of treatment).

**Table 2:** PATRO Adult effectiveness parameters by pre-treatment status

Visit (years)	Mean (SD) IGF-I SDS (EFF)		Mean (SD) waist (cm) (EFF)		Mean (SD) BMI (kg/m^**2**^) (EFF)		Mean (SD) HDL/LDL ratio (SAF)		Mean (SD) rhGH dose (mg/day) (EFF)	
	Naïve	Pre-treated	Naïve	Pre-treated	Naïve	Pre-treated	Naïve	Pre-treated	Naïve	Pre-treated
Baseline	−3.0 (1.4) *n =* 10	−0.1 (1.6) *n =* 35	100.6 (13.6) *n =* 85	96.9 (13.3) *n =* 107	29.1 (5.6) *n =* 111	28.1 (5.4) *n =* 130	3.1 (1.4) *n =* 18	2.3 (1.1) *n =* 83	0.1 (0.1) *n =* 120	0.4 (0.3) *n =* 146
1	−1.2 (2.0) *n =* 9	0.5 (1.2) *n =* 8	98.9 (12.9) *n =* 35	96.0 (11.8) *n =* 64	29.4 (5.0) *n =* 56	27.8 (4.7) *n =* 77	2.6 (1.1) *n =* 25	2.3 (1.1) *n =* 62	0.3 (0.2)^a^ *n =* 114	0.4 (0.2)^a^ *n =* 142
2	−0.8 (1.6) *n =* 7	1.8 (1.3) *n =* 2	99.4 (13.0) *n =* 23	94.5 (11.3) *n =* 66	29.1 (5.5) *n =* 32	27.2 (4.4) *n =* 76	2.5 (0.5) *n =* 11	2.2 (0.9) *n =* 46	0.3 (0.2)^b^ *n =* 98	0.4 (0.2)^b^ *n =* 133
3	0.0 (2.4) *n =* 2	1.8 (1.0) *n =* 3	98.2 (15.0) *n =* 15	95.3 (12.0) *n =* 51	28.8 (6.7) *n =* 22	27.8 (6.0) *n =* 57	2.3 (0.6) *n =* 10	2.2 (1.0) *n =* 38	0.3 (0.2)^c^ *n =* 72	0.4 (0.2)^c^ *n =* 117
4	−0.8 (1.7) *n =* 6	−0.2 (3.6) *n =* 2	102.3 (11.7) *n =* 15	97.6 (17.0) *n =* 34	30.0 (7.6) *n =* 23	28.0 (5.3) *n =* 40	2.2 (0.6) *n =* 11	2.1 (0.8) *n =* 25	0.3 (0.2)^d^ *n =* 58	0.3 (0.2)^d^ *n =* 96

*n* Number of patients with available data

*BMI* Body mass index, *EFF* Effectiveness analysis set, *IGF-I* Insulin-like growth factor, *HDL* High-density lipoprotein, *LDL* Low-density lipoprotein, *PATRO* Patients TReated with Omnitrope®, *rhGH* Recombinant human growth hormone, *SAF* Safety analysis set, *SD(S)* Standard deviation (score)

^a^Daily dose at years 1–1.5; ^b^Daily dose at years 2–2.5; ^c^Daily dose at years 3–3.5; ^d^Daily dose at years 4–4.5

Mean BMI and waist circumference were higher at baseline and during rhGH treatment in rhGH-naïve patients compared with pre-treated patients. After the first year of rhGH therapy, IGF-I SDS was higher in pre-treated patients, who also received higher rhGH doses throughout the study period (Table 2).

## Discussion

Omnitrope® is the world’s first approved biosimilar rhGH and was introduced in Sweden in 2007. This report describes over 10 years of treatment experience in Sweden, collected in routine clinical practice as part of the observational PATRO studies. Until 2016, Swedish data was included in international reports but here we have added three more years of observation and more detailed, individual analyses.

The main aim of the PATRO studies is to follow patients for safety concerns such as the occurrence of diabetes mellitus or malignancies, and to detect anti-hGH antibodies in rhGH treatment-naïve children. The outcomes we report over 10 years are reassuring in terms of safety and effectiveness across both pediatric and adult indications.

In PATRO Children, no diabetes mellitus, impaired glucose tolerance, or malignancy were reported. The most frequent AEs were of mild intensity and did not require discontinuation of rhGH therapy. These findings are consistent with data from previous studies [9, 16]. Immunogenicity is a potential risk for biological pharmaceuticals, including rhGH [17], and in theory, the clinical consequence of antibody development might include a loss of efficacy [18]. The low (i.e. absent) incidence of antibody formation against biosimilar rhGH in the Swedish PATRO Children population confirms earlier results from interventional studies of children with GHD [16, 19] and is consistent with earlier data from PATRO Children, which included preliminary Swedish data [9, 20].

The main reasons for changing from rhGH to biosimilar rhGH for pediatric patients were economic. A change of medicine and injection device may require re-education of patients and families in rhGH administration, an often time-consuming process. This could result in inconvenience or even have negative effects on efficacy if adherence is affected, or patients or family members have safety concerns relating to a change in medication [21]. However, a new injection device may improve compliance due to greater patient acceptability [22]. In our study, we did not see any negative effects associated with changing to biosimilar rhGH, and reported AEs were mild, supporting earlier analyses that found switching to biosimilar rhGH had no impact on safety [23, 24].

In PATRO Adults, development of neoplasms and diabetes mellitus was reported in 26 and 4 patients, respectively. Of note, a relationship between development of cancer (in 8 patients) or relapse of pituitary tumor and rhGH treatment was not suspected by the treating physician or study sponsor for any of the patients, consistent with observations in a previous review [25]. Regarding the onset of diabetes mellitus, rhGH treatment might impair glucose metabolism, especially in obese patients with a family history of diabetes mellitus. However, the prospective KIMS (Pfizer International Metabolic Database) and NordiNet (Novo Nordisk) registers of non-biosimilar rhGH-treated adults, have reported no increase in the incidence of diabetes mellitus type 2 in patients with GHD and a normal BMI, although an increase in fasting blood glucose (within normal limits) is frequently reported [25, 26]. In PATRO Adults, AEs were mild in severity, transient, and related to the dose of biosimilar rhGH. Frequently observed side effects with rhGH treatment, such as edema, muscle pain, joint stiffness and pain, paraesthesia, carpal tunnel syndrome, and headache, are often caused by fluid retention [25, 26]. Our present safety data from Swedish patients enrolled in the PATRO Adults study were consistent with previous observations and reports [25, 26] as most AEs occurred early and were related to fluid retention.

Biosimilar rhGH was effective in the pediatric population, although only a few rhGH-naïve patients attained AH or had reached the pubertal period by the January 2019 interim analysis. Further evaluation of effectiveness will be possible when more patients have completed the study at age 18 years. Among the six patients reported as non-responders, there were no signs of the biosimilar rhGH being ineffective. Only two of the six patients, diagnosed with familial short stature and hypochondroplasia, respectively, could be judged as genuine non-responders according to the Swedish recommendation of a first-year gain in HSDS growth response < 0.5, despite treatment with an adequate rhGH dose within label [27]. Familial short stature needs careful evaluation, as a good response to rhGH therapy is rarely seen [28–30], a situation that is similar to short patients with fetal alcohol syndrome [31]. Height velocity may be improved in children with hypochondroplasia with rhGH doses averaging 0.053 (range, 0.022–0.086) mg/kg/day, but responses vary [32, 33]. Four of the children with low growth response received a lower than recommended rhGH dose. The International TS Consensus Group recommends an initial dose 0.045–0.050 mg/kg/day, increasing to 0.068 mg/kg/day if AH potential is compromised [34]. For short children born SGA, the range for effect on growth is reported to be wide [35]. The reported rhGH doses in PATRO Children were lower on average compared with the recommended doses for similar patient groups worldwide [1, 27]. The Swedish recommendation for rhGH dose in GHD patients is 0.033 mg/kg/day based on studies of the physiology of GH secretion in children [28]. In Swedish national randomized clinical trials, an rhGH dose of 0.067 mg/kg/day during puberty showed higher growth responses compared with standard doses in children diagnosed with GHD or non-GHD short stature [29, 30]. In PATRO, use of rhGH doses below those recommended may be explained by caution among physicians in the use of a clinically new drug. In addition, during the introduction of the biosimilar rhGH, a report from the French SAGhE (Safety and Appropriateness of Growth Hormone Treatments in Europe) study suggested an increased risk of mortality due to bone tumors and cerebrovascular diseases in patients with GHD, ISS, or SGA treated with rhGH during childhood [31]. Swedish, Belgian, and Dutch data from the same period did not support this finding [32]; instead it was shown that size at birth is an important factor [33]. However, concerns about the consequences of rhGH dosage remain [1, 34], and the consensus is to use the lowest effective dose if therapy is started.

Another reason for using reduced rhGH doses in the PATRO Children study may be that individual rhGH responsiveness was not considered [35]. In group analyses, patients with non-GHD-related short stature need a higher dose to attain the same growth response as patients with GHD [36]. This was shown for prepubertal patients [36] and was later confirmed for pubertal growth until AH [29, 30, 37, 38]. The variation in growth response in the SGA group could possibly have been reduced by estimation of responsiveness using a prediction model [39, 40].

Adult GHD is characterized by abnormal body composition, with more body fat than lean body mass, an adverse lipid profile, and poor quality of life [2]. Several studies have shown that rhGH treatment improves these variables [2, 26, 41]. Baseline characteristics for patients in PATRO Adults were similar to previous reports of adults with GHD (i.e. low IGF-I, overweight, and elevated waist circumference) [2, 26, 41]. Also, consistent with previous studies, we found that BMI and waist size remained stable during treatment with biosimilar rhGH [2, 26, 41]. Previous studies [2, 26, 41] have shown a decrease in total and LDL cholesterol, and in agreement with these findings we also observed a decrease in LDL/HDL cholesterol ratio.

### Strengths and limitations

A strength of the study is the comprehensive and uniform long-term follow up. Data collected are monitored twice a year by a contract research organization to ensure accuracy. Females represent 50% of the population in PATRO Children, with or without the TS group. This is a positive trend compared with earlier studies from the Swedish national GH register and from Swedish clinical trials [29, 30].

One limitation of the pediatric part of the study is the short observation time to detect growth until AH. Additionally, the small number of patients with different diagnoses and GHD etiologies results in a heterogeneous cohort, preventing sub-group analyses. It is also a limitation that blood samples were not analyzed centrally. Furthermore, there is a potential selection bias due to enrolment of patients from a few centers. In addition, it was not possible to compare the incidence of AEs reported in the study with the background rate occurring in a Swedish population. Systematic follow up in national databases will improve control, research, and individualization of rhGH treatment.

## Conclusion

This is the first report of long-term results from the observational PATRO studies of the first biosimilar rhGH, presenting results for the Swedish children and adult cohorts. Based on over 10 years of observation in real-life clinical practice, the available data suggest that biosimilar rhGH is well tolerated and effective across pediatric and adult indications. No increased risk was seen for neoplasia, diabetes mellitus, or immunogenicity compared with other rhGH treatments. Effectiveness and safety were similar to previously reported data for other rhGH preparations. However, our results need to be confirmed over longer durations and in larger cohorts, incorporating a comparison with the background rate of reported AEs in the overall population.

## Data Availability

The datasets generated during and/or analyzed during the current study are not publicly available as the study is still ongoing, but are available from the corresponding author on reasonable request.

## Abbreviations

125I: Radio isotope labeled
AE: Adverse event
AH: Adult height
BMI: Body mass index
CRI: Chronic renal insufficiency
eCRF: Electronical case record form
EFF: Effectiveness analysis set
EMA: European Medical Agency
GH: Growth hormone
GHD: Growth hormone deficiency
HDL: High-density lipoprotein
IGF-I: Insulin-like growth factor-I
ISS: Idiopathic short stature
LDL: Low-density lipoprotein
PATRO: Patients TReated with Omnitrope®
PWS: Prader–Willi syndrome
rhGH: Recombinant human growth hormone
SAE: Serious AE
SAF: Safety analysis set
SD: Standard deviation
SDS: Standard deviation score
SGA: Small for gestational age
TS: Turner syndrome

## References

[1] Society GHR (2000). Consensus guidelines for the diagnosis and treatment of growth hormone (GH) deficiency in childhood and adolescence: summary statement of the GH research Society. GH research Society. J Clin Endocrinol Metab.

[2] Ho KK (2007). Consensus guidelines for the diagnosis and treatment of adults with GH deficiency II: a statement of the GH research Society in association with the European Society for Pediatric Endocrinology, Lawson Wilkins Society, European Society of Endocrinology, Japan Endocrine Society, and Endocrine Society of Australia. Eur J Endocrinol.

[3] Schellekens H (2002). Bioequivalence and the immunogenicity of biopharmaceuticals. Nat Rev Drug Discov.

[4] Roger SD, Mikhail A (2007). Biosimilars: opportunity or cause for concern. J Pharm Pharm Sci.

[5] Kessler M, Goldsmith D, Schellekens H (2006). Immunogenicity of biopharmaceuticals. Nephrol Dial Transplant.

[6] Saenger P (2009). Current status of biosimilar growth hormone. Int J Pediatr Endocrinol.

[7] European Medicines Agency (2014). Guideline on similar biological medicinal products. Committee for Medicinal Products for Human Use.

[8] Beck-Peccoz P, Minuto F, Leal-Cerro A, Zabransky M, Stalla G (2012). Rationale and design of PATRO adults, a multicentre, noninterventional study of the long-term efficacy and safety of Omnitrope® for the treatment of adult patients with growth hormone deficiency. Ther Adv Endocrinol Metab.

[9] Pfäffle R, Schwab KO, Marginean O, Walczak M, Szalecki M, Schuck E (2013). Design of, and first data from, PATRO children, a multicentre, noninterventional study of the long-term efficacy and safety of Omnitrope® in children requiring growth hormone treatment. Ther Adv Endocrinol Metab.

[10] Albertsson-Wikland K, Luo ZC, Niklasson A, Karlberg J (2002). Swedish population-based longitudinal reference values from birth to 18 years of age for height, weight and head circumference. Acta Paediatr.

[11] Karlberg J (1989). On the construction of the infancy-childhood-puberty growth standard. Acta Paediatr Scand Suppl.

[12] He Q, Albertsson-Wikland K, Karlberg J (2000). Population-based body mass index reference values from Göteborg, Sweden: birth to 18 years of age. Acta Paediatr.

[13] Tanner J, Whitehouse R (1976). Clinical longitudinal standards for height, weight, height velocity, weight velocity, and stages of puberty. Arch Dis Child.

[14] Prader A (1966). Testicular size: assessment and clinical importance. Triangle..

[15] Friedewald WT, Levy RI, Fredrickson DS (1972). Estimation of the concentration of low density lipoprotein cholesterol in plasma, without the use of the preparative ultracentrifuge. Clin Chem.

[16] Romer T, Saenger P, Peter F, Walczak M, Le Bouc Y, Khan-Boluki J (2009). Seven years of safety and efficacy of the recombinant human growth hormone Omnitrope in the treatment of growth hormone deficient children: results of a phase III study. Horm Res Paediatr.

[17] Ryff J-C, Schellekens H (2002). Immunogenicity of rDNA-derived pharmaceuticals. Trends Pharmacol Sci.

[18] Schellekens H (2009). Biosimilar therapeutics—what do we need to consider?. NDT Plus.

[19] Lopez-Siguero J, Borras Perez MV, Balser S, Khan-Boluki J (2011). Long-term safety and efficacy of the recombinant human growth hormone Omnitrope® in the treatment of Spanish growth hormone deficient children: results of a phase III study. Adv Ther.

[20] Pérez MVB, Kriström B, Romer T, Walczak M, Höbel N, Zabransky M (2017). Ten years of clinical experience with biosimilar human growth hormone: a review of safety data. Drug Des Dev Ther.

[21] Rashid N, Saenger P, Wu Y-L, Woehling H, Frankel M, Lifshitz F (2014). Switching to Omnitrope® from other recombinant human growth hormone therapies: a retrospective study in an integrated healthcare system. Biol Ther.

[22] Coutant R, Dupuis C, Pigeon P, Rebaud P (2017). Patients’ perceptions on the usability of the SurePal™ self-injection device for Omnitrope®: a questionnaire-based observational study conducted in paediatric patients in France. Ther Adv Endocrinol Metab.

[23] Romer T, Zabransky M, Walczak M, Szalecki M, Balser S (2011). Effect of switching recombinant human growth hormone: comparative analysis of phase 3 clinical data. Biol Ther.

[24] Flodmark C-E, Lilja K, Woehling H, Järvholm K (2013). Switching from originator to biosimilar human growth hormone using dialogue teamwork: single-center experience from Sweden. Biol Ther.

[25] Stochholm K, Kiess W (2018). Long-term safety of growth hormone—a combined registry analysis. Clin Endocrinol.

[26] Höybye C, Christiansen JS (2015). Growth hormone replacement in adults–current standards and new perspectives. Best Pract Res Clin Endocrinol Metab.

[27] Gravholt CH, Andersen NH, Conway GS, Dekkers OM, Geffner ME, Klein KO (2017). Clinical practice guidelines for the care of girls and women with turner syndrome: proceedings from the 2016 Cincinnati international turner syndrome meeting. Eur J Endocrinol.

[28] Albertsson-Wikland K, Rosberg S, Libre E, Lundberg LO, Groth T (1989). Growth hormone secretory rates in children as estimated by deconvolution analysis of 24-h plasma concentration profiles. Am J Phys.

[29] Albertsson-Wikland K, Aronson AS, Gustafsson J, Hagenäs L, Ivarsson SA, Jonsson B (2008). Dose-dependent effect of growth hormone on final height in children with short stature without growth hormone deficiency. J Clin Endocrinol Metab.

[30] Albertsson-Wikland K, Kristrom B, Lundberg E, Aronson AS, Gustafsson J, Hagenas L (2014). Growth hormone dose-dependent pubertal growth: a randomized trial in short children with low growth hormone secretion. Horm Res Paediatr.

[31] Carel J-C, Ecosse E, Landier F, Meguellati-Hakkas D, Kaguelidou F, Rey G (2012). Long-term mortality after recombinant growth hormone treatment for isolated growth hormone deficiency or childhood short stature: preliminary report of the French SAGhE study. J Clin Endocrinol Metab.

[32] Sävendahl L, Maes M, Albertsson-Wikland K, Borgström B, Carel J-C, Henrard S (2012). Long-term mortality and causes of death in isolated GHD, ISS, and SGA patients treated with recombinant growth hormone during childhood in Belgium, the Netherlands, and Sweden: preliminary report of 3 countries participating in the EU SAGhE study. J Clin Endocrinol Metab.

[33] Albertsson-Wikland K, Mårtensson A, Sävendahl L, Niklasson A, Bang P, Dahlgren J (2016). Mortality is not increased in recombinant human growth hormone-treated patients when adjusting for birth characteristics. J Clin Endocrinol Metab.

[34] Wilson TA, Rose SR, Cohen P, Rogol AD, Backeljauw P, Brown R (2003). Update of guidelines for the use of growth hormone in children: the Lawson Wilkins pediatric endocrinology Society drug and therapeutics committee. J Pediatr.

[35] Kriström B, Dahlgren J, Niklasson A, Nierop AF, Albertsson-Wikland K (2009). The first-year growth response to growth hormone treatment predicts the long-term prepubertal growth response in children. BMC Med Inform Decis Mak.

[36] Kriström B, Aronson AS, Dahlgren J, Gustafsson J, Halldin M, Ivarsson SA (2009). Growth hormone (GH) dosing during catch-up growth guided by individual responsiveness decreases growth response variability in prepubertal children with GH deficiency or idiopathic short stature. J Clin Endocrinol Metab.

[37] Mauras N, Attie KM, Reiter EO, Saenger P, Baptista J (2000). High dose recombinant human growth hormone (GH) treatment of GH-deficient patients in puberty increases near-final height: a randomized, multicenter trial. Genentech, Inc., cooperative study group. J Clin Endocrinol Metab.

[38] Sas TC, de Ridder MA, Wit JM, Rotteveel J, Oostdijk W, Reeser HM (2010). Adult height in children with growth hormone deficiency: a randomized, controlled, growth hormone dose-response trial. Horm Res Paediatr.

[39] Ranke MB, Lindberg A, Cowell CT, Wikland KA, Reiter EO, Wilton P (2003). Prediction of response to growth hormone treatment in short children born small for gestational age: analysis of data from KIGS (Pharmacia international growth database). J Clin Endocrinol Metab.

[40] Dahlgren J, Kriström B, Niklasson A, Nierop AF, Rosberg S, Albertsson-Wikland K (2007). Models predicting the growth response to growth hormone treatment in short children independent of GH status, birth size and gestational age. BMC Med Inform Decis Mak.

[41] Molitch ME, Clemmons DR, Malozowski S, Merriam GR, Vance ML (2011). Evaluation and treatment of adult growth hormone deficiency: an Endocrine Society clinical practice guideline. J Clin Endocrinol Metab.

